# Efficacy and safety of the long-acting C5 inhibitor ravulizumab in patients with atypical hemolytic uremic syndrome triggered by pregnancy: a subgroup analysis

**DOI:** 10.1186/s12882-020-02190-0

**Published:** 2021-01-06

**Authors:** Anja Gäckler, Ulf Schönermarck, Vladimir Dobronravov, Gaetano La Manna, Andrew Denker, Peng Liu, Maria Vinogradova, Sung-Soo Yoon, Manuel Praga

**Affiliations:** 1 Department of Nephrology, University Hospital Essen, University Duisburg-Essen, Essen, Germany; 2 Medizinische Klinik IV, LMU Klinikum, LMU, Munich, Germany; 3 Pavlov University, Research Institute of Nephrology, St. Petersburg, Russia; 4 Department of Experimental Diagnostic and Specialty Medicine (DIMES), Nephrology, Dialysis and Renal Transplant Unit, St. Orsola Hospital, University of Bologna, Bologna, Italy; 5 Alexion Pharmaceuticals, Inc., Boston, USA; 6 National Medical Research Centre for Obstetrics and Gynecology, Moscow, Russia; 7 Seoul National University Hospital, Seoul, Republic of Korea; 8 Instituto de Investigación Hospital Universitario 12 de Octubre i+12, Madrid, Spain

**Keywords:** Ravulizumab, Thrombotic microangiopathy, Pregnancy microangiopathies, Atypical hemolytic uremic syndrome

## Abstract

**Background:**

Atypical hemolytic uremic syndrome (aHUS) triggered by pregnancy is a rare disease caused by dysregulation of the alternative complement pathway that occurs in approximately 1 in 25,000 pregnancies. The 311 phase 3 trial (NCT02949128) showed that ravulizumab, a long-acting C5 inhibitor obtained through selective modifications to eculizumab, is efficacious in inhibiting complement-mediated thrombotic microangiopathy (TMA) in patients with aHUS. In this analysis, we report outcomes in a subgroup of patients from the 311 study who developed TMA postpartum.

**Methods:**

This was a phase 3, multicenter trial evaluating efficacy and safety of ravulizumab in adults (≥18 years of age) with aHUS naïve to complement inhibitor treatment. The primary endpoint was complete TMA response (simultaneous platelet count normalization [≥150 × 10^9^/L], lactate dehydrogenase normalization [≤246 U/L] and 25% improvement in serum creatinine) through the 183-day initial evaluation period. Additional efficacy endpoints included time to complete TMA response, hematologic normalization, and dialysis requirement status.

**Results:**

Eight patients presenting with TMA postpartum (median age of 37.7 [range; 22.1–45.2] years) were diagnosed with aHUS and received ≥1 dose of ravulizumab. Five patients (63%) were on dialysis at baseline. Complete TMA response was achieved in 7/8 patients (87.5%) in a median time of 31.5 days. Hematologic normalization was observed in all patients. All patients on dialysis at baseline discontinued dialysis within 21 days after treatment with ravulizumab. All patients showed continued improvements in the estimated glomerular filtration rate from baseline to Day 183. Three possible treatment-related adverse events were observed in 2 patients (arthralgia and nasopharyngitis [both non-severe]; urinary tract infection). No deaths or meningococcal infections occurred.

**Conclusions:**

Treatment with ravulizumab provided immediate and complete C5 inhibition, resulting in rapid clinical and laboratory improvements and complete TMA response through 183 days in patients with aHUS triggered by pregnancy. The safety profile observed in this subset of patients analysed is consistent with the 311 study investigating ravulizumab in patients with aHUS naïve to complement treatment.

**Trial registration:**

**Clinical trial identifier:** NCT02949128.

**Supplementary Information:**

The online version contains supplementary material available at 10.1186/s12882-020-02190-0.

## Background

Atypical hemolytic uremic syndrome (aHUS) is a rare, life-threatening disease caused by dysregulation of the alternative complement pathway, presenting as thrombotic microangiopathy (TMA; hemolytic anemia, thrombocytopenia, and organ injury – usually the kidney) [1, 2]. Over the past few years, there is increasing consensus that, in the majority of patients, aHUS may involve both genetic predisposition (e.g., pathogenic variants, autoantibodies or at-risk polymorphisms in complement genes) and a triggering condition in order for the clinical event of a TMA to occur [3–5]. However, a genetic predisposition is not always identified and is not required for diagnosis.

Pregnancy is a known triggering condition for the manifestation of the disease. aHUS triggered by pregnancy is a rare and under-recognized complement-mediated TMA [5], occurring in approximately 1 in 25,000 pregnancies [6] and in 4% of diagnosed cases of aHUS [3]. The onset of aHUS triggered by pregnancy is more common in the postpartum period (79%) [7], but can also occur in the peripartum period. Diagnosis is often difficult because aHUS shares similar clinical features to other syndromes triggered by pregnancy, including ADAMTS13 (a disintegrin and metalloproteinase with a thrombospondin type 1 motif, member 13) deficiency-associated thrombotic thrombocytopenic purpura (TTP), pre-eclampsia, and HELLP (hemolysis, elevated liver enzymes and low platelets) syndrome [1].

aHUS triggered by pregnancy is considered a medical emergency, requiring hospitalization and prompt initiation of appropriate treatment for optimal maternal outcomes. Overall, the disease course in aHUS triggered by pregnancy and aHUS not related to pregnancy is similar, with some studies reporting that two-thirds of patients require dialysis at onset, and more than half reach end-stage renal disease (ESRD) within 1 month of onset [8], despite treatment with corticosteroids or plasma exchange [9]. Generally, outcomes for patients with aHUS triggered by pregnancy are poor without terminal complement inhibitor treatment [10].

Eculizumab (Alexion Pharmaceuticals, Inc., Boston, MA, USA), a humanized monoclonal antibody that blocks terminal complement activation at C5 [11, 12], has significantly improved the clinical outcomes in patients with aHUS. The efficacy and safety profile of eculizumab has been demonstrated across 4 prospective clinical trials [13–17], registry and non-trial patient data [8, 18], including studies on patients with aHUS triggered by pregnancy [8, 19–21]. Ravulizumab (Alexion Pharmaceuticals, Inc., Boston, MA, USA) is a new long-acting monoclonal antibody obtained through selective modifications to eculizumab, allowing extended maintenance dosing from every 2 to every 8 weeks [22]. Ravulizumab was recently approved for the treatment of adults and children with aHUS in the United States [23].

The phase 3 ALXN1210-aHUS-311 clinical trial (hereon referred to as ‘311’) demonstrated the efficacy and safety profile in a cohort of 56 adults with aHUS naïve to complement inhibitor therapy treated for acute TMA [24]. The purpose of this analysis is to report both clinical characteristics and outcomes in a subset of 8 patients with aHUS from the 311 study who presented postpartum and received ravulizumab. This is the largest cohort of patients from a clinical trial to evaluate the efficacy and safety of C5 inhibitors in postpartum aHUS.

## Methods

### Trial oversight and study design

The 311 clinical trial (NCT02949128) is a phase 3, single arm, multicenter study designed to evaluate the efficacy and safety of ravulizumab administered by intravenous (IV) infusion to adults with aHUS naïve to complement inhibitor treatment. Patients from the trial included in this analysis were ≥18 years of age and weighed ≥40 kg with active TMA (thrombocytopenia, evidence of hemolysis and kidney dysfunction) at postpartum. All patients included in this analysis had evidence of TMA lasting for ≥3 days. Patients could meet the platelet and lactate dehydrogenase (LDH) criteria (<150 × 10^9^ and ≥1.5 x upper limit of normal, respectively) based on results from local laboratories, but the serum creatinine criteria (≥ upper limit of normal) must have been confirmed by the central laboratory at baseline.

The study consisted of an initial evaluation period of 183 days. Ravulizumab was administered via IV loading dose of 2400 mg, 2700 mg or 3000 mg in patients weighing ≥40–< 60 kg, ≥60–<100 kg, or ≥100 kg, respectively, on Day 1. Maintenance doses of 3000 mg, 3300 mg, 3600 mg, respectively, were administered on Day 15 and then every 8 weeks thereafter. Baseline was defined as the period of screening up to before the point of the first study drug infusion, including Day 1 (study design described previously in detail [24, 25]).

Patients must have received meningococcal vaccination according to local and national guidelines at the time of commencing therapy and were also required to receive antibiotic prophylaxis from the time of first dose of ravulizumab until at least 2 weeks after vaccination.

Patients with ADAMTS13 deficiency (activity <5%); Shiga toxin-producing *Escherichia coli*-HUS; hematopoietic stem cell transplantation in the 6 months prior to screening and history of malignancy within 5 years of screening were excluded. Patients who had received complement inhibitors, immunosuppressive therapies (except for kidney transplant regimens), steroids, patients who received tranexamic acid within 7 days, and patients on chronic dialysis were also excluded. Plasma exchange/infusion (PE/PI) was allowed up to, but not after, the first dose of ravulizumab, but patients were excluded if therapy exceeded 28 days.

The protocol was approved by the Institutional Review Board or Independent Ethics Committee at each participating center, and the study was conducted in accordance with the Declaration of Helsinki and the Council for International Organizations of Medical Sciences International Ethical Guidelines. Written informed consent was obtained from all individual participants or legal guardians, as applicable.

### Efficacy and safety endpoints

Full details of the efficacy and safety endpoints can be found in the primary analysis publication [24]. The primary efficacy endpoint was complete TMA response through an initial evaluation period of 183 days. The criteria for complete TMA response were platelet count normalization (≥150 × 10^9^/L), LDH normalization (≤246 U/L) and ≥25% improvement in serum creatinine from baseline met concurrently and at two separate assessments ≥28 days apart, and any measurement in between. When a patient was on dialysis at baseline, the baseline value used for serum creatinine response assessment was the first value at 6 or more days post-dialysis. Patients were considered as being on dialysis at baseline if dialysis occurred within 5 days prior to ravulizumab initiation.

Secondary objectives of the study included time to complete TMA response; change in hematologic variables (platelets, LDH, and hemoglobin); change in estimated glomerular filtration rate (eGFR) values; and dialysis requirement status. Exploratory genetic analysis by whole exome sequencing was conducted on patients in the original study. Additional genetic analyses performed at the centers treating the individual patients in this analysis were also included. Safety and tolerability of ravulizumab were evaluated by clinical and laboratory assessment and frequency of adverse events (AEs) and serious AEs (SAE). Full details on the study methodology have been previously described in detail in the primary analysis [24].

## Results

### Patient characteristics

Eight postpartum patients with a median age of 37.7 (range; 22.1–45.2) years met the inclusion criteria, were enrolled and received ≥1 dose of ravulizumab. None of the patients reported breastfeeding during the study. Baseline demographics and disease characteristics are shown in Table 1.

**Table 1:** Baseline demographics and disease characteristics

Variable	Overall (***N*** = 8)
Median (min, max) age at first infusion, years	37.7 (22.1, 45.2)
Age at time of first infusion (years) category	
18 to <30 years	2 (25.0)
30 to <40 years	3 (37.5)
40 to <50 years	3 (37.5)
Race	
Asian	1 (12.5)
White	7 (87.5)
ADAMTS13 activity >5%	8 (100)
Extrarenal signs or symptoms of aHUS prior to first infusion of drug	6 (75)
Baseline laboratory values, median (min, max) ^a^	
Platelet count, ×10^9^/L	119 (36, 473)
LDH, U/L	576 (280, 876)
Serum creatinine, μmol/L	408 (51, 758)
HGB, g/L	72.8 (63, 105.5)
eGFR, mL/min/1.73 m^2(b)^	10.0 (10, 18)
Median (min, max) time from delivery to first dose, days	11 (5, 19)
ICU care required	7 (87.5)
Median (min, max) stay in ICU, days	9 (2, 21)
Received PE/PI related to this TMA prior to first infusion of drug	6 (75)
On dialysis within 5 days of first dose	5 (62.5)
Median (min, max) time on dialysis prior to first dose, days	5 (4, 8)
Patients with ≥1 identified pathogenic variant or autoantibody	2 (25)
CFB pathogenic variant	1 (12.5)
Anti-CFH antibodies	1 (12.5)
None identified	5 (65.5)
Data not available	1 (12.5)

^a^Baseline values may be after PE/PI in some patients. ^b^eGFR in patients on dialysis was set to 10 mL/min/1.73 m^2^, and eGFR was calculated using the Modification of Diet in Renal Disease formula. Data displayed as n (%) unless otherwise stated

*ADAMTS13* a disintegrin and metalloproteinase with a thrombospondin type 1 motif, member 13, *aHUS* atypical hemolytic uremic syndrome, *CFB* complement factor B, *CFH* complement factor H, *eGFR* estimated glomerular filtration rate, *HGB* hemoglobin, *ICU* intensive care unit, *LDH* lactate dehydrogenase, *PE* plasma exchange, *PI* plasma infusion, *TMA* thrombotic microangiopathy

At baseline, and prior to any ravulizumab dose, all patients presented with acute, severe medical emergency associated with the pregnancy or delivery. All patients had complicated deliveries and most also had antenatal complications. Pre-eclampsia and hypertension were reported in 2 patients each, and renal failure and gestational diabetes in 1 patient each. Two patients suffered placental abruption, resulting in antenatal fetal death in 1 of these cases. Five patients underwent emergency cesarean section with complications occurring in 4 cases; hemorrhage in 2, secondary hysterectomy in 2 (1 with hemorrhage) and fetal death in 1 case. Additional data on the events prior to TMA are detailed in Supplementary Table 1.

All patients completed the 183-day initial evaluation period with no study or drug discontinuations.

### Primary endpoint

**Fig. 1:**
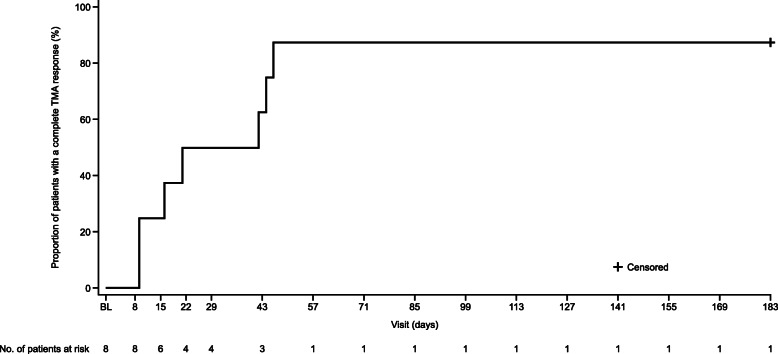
Kaplan–Meier graph depicting time to complete TMA response. *BL* baseline, *TMA* thrombotic microangiopathy

During the initial evaluation period, 7 of 8 patients (87.5%) met the primary endpoint of complete TMA response (Fig. 1). The patient that did not achieve complete TMA response had a rapid response to ravulizumab treatment, with normalization of both platelets and LDH on Day 8. She had a dialysis session 5 days before first dose, and baseline creatinine was the value obtained on Day 8 (≥6 days after last dialysis session as defined by protocol). On Day 8, this patient had already shown improved serum creatinine levels to 51 μmol/L, which is well within the normal range, and an additional improvement of 25% in serum creatinine from this value was not reached, which would have been the requirement to meet complete TMA response criteria.

### Secondary endpoints

**Fig. 2:**
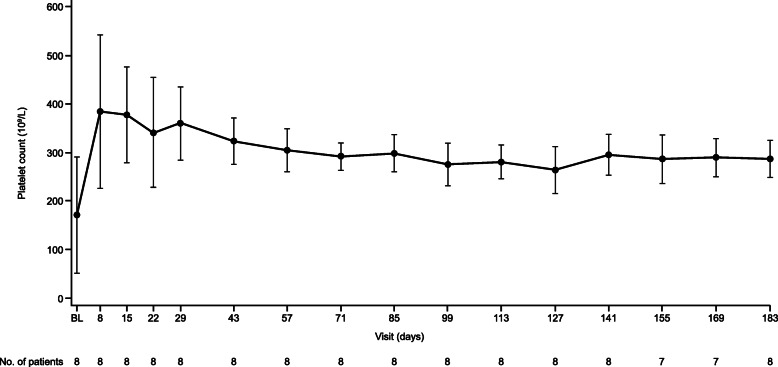
Observed platelet count value over time. Data are shown as mean (error bars, 95% confidence interval). *BL* baseline

**Fig. 3:**
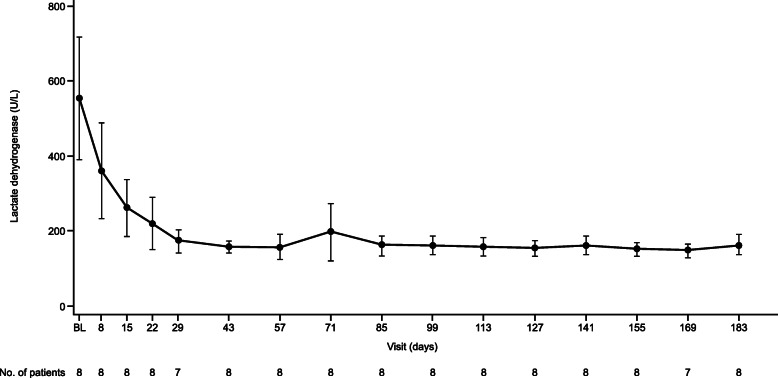
Observed lactate dehydrogenase values over time. Data are shown as mean (error bars, 95% confidence interval). *BL* baseline

**Fig. 4:**
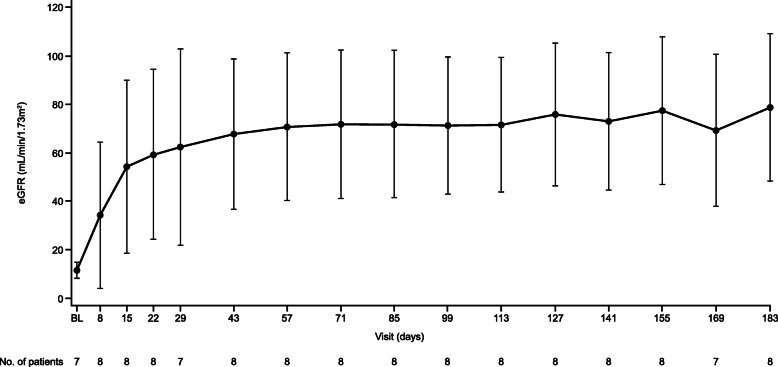
Observed eGFR values over time. Data are shown as mean (error bars, 95% confidence interval). *BL* baseline, *eGFR* estimated glomerular filtration rate

The median (95% CI) time to complete TMA response was 31.5 (9.0, 46.0) days (Fig. 1). Hematologic normalization, platelet count and LDH normalization were observed in all patients (100%). Platelet count, LDH and eGFR all improved rapidly (Fig. 2, Fig. 3, Fig. 4). All patients on dialysis at baseline were able to discontinue dialysis within 21 days of commencing treatment with ravulizumab.

### Safety

Adverse events post-treatment initiation were observed in all 8 patients included in the analysis (Table 2). The most common adverse events reported in at least 2 patients were headache and pyrexia (occurring in 3 patients each). Other adverse events reported in 2 patients each included constipation, urinary tract infection, nasopharyngitis, alopecia, hypertension, arthralgia, increased alanine aminotransferase, and increased aspartate aminotransferase. Three possible treatment-related adverse events (as determined by the investigator) were noted in 2 patients (arthralgia and nasopharyngitis [both non-severe]; urinary tract infection). Both patients recovered from these events. One serious adverse event was reported. This event was a routine renal biopsy unrelated to treatment with ravulizumab. No deaths or meningococcal infections occurred.

**Table 2:** Summary of adverse events

	Overall (***N*** = 8)	
AE Categories	***n*** (%)	Events
Any AE	8 (100.0)	71
Any SAE	1 (12.5)	1
Fatal TEAEs	0 (0.0)	0
TEAEs or TESAEs resulting in drug discontinuation	0 (0.0)	0
TEAEs or TESAEs resulting in study discontinuation	0 (0.0)	0
Meningococcal infections	0 (0.0)	0
Treatment-related AEs (all considered possibly-related)	2 (25.0)	3

*AE* adverse event, *SAE* serious adverse event, *TEAE* treatment-emergent adverse event, *TESAE* treatment-emergent serious adverse event

## Discussion

This is the largest cohort of patients from a prospective interventional study to evaluate the efficacy and safety of C5 inhibitors in postpartum aHUS. All patients presented in a severe condition; 62.5% of patients required dialysis at baseline and 87.5% of patients required ICU level care, similar to other reports detailing patients with aHUS triggered by pregnancy [7]. All patients responded rapidly to treatment with ravulizumab, with a median time to complete TMA response of 31.5 days. Although one patient did not achieve the primary endpoint of TMA response, this patient had a rapid response to ravulizumab treatment, with normalization of both platelets and LDH by Day 8. Serum creatinine levels were within normal range on Day 8, thus the patient could not achieve a 25% improvement in serum creatinine levels required to fulfil the primary endpoint. No safety concerns were identified in this study.

The data obtained in this subset analysis show that a higher proportion of patients presenting postpartum resolved TMA with ravulizumab treatment compared to the full cohort of patients with aHUS in the 311 study [24]. Patients in this analysis received ravulizumab treatment soon after delivery (median 11 [range, 5–19] days), whereas the time to treatment in the overall 311 study was broader, with patients receiving their first dose of ravulizumab as early as at the onset of symptoms or as late as 215 months after the first symptom of aHUS, highlighting the importance of early treatment. Moreover, the median time to complete TMA response in this subgroup of patients was shorter than the full cohort in the 311 study (31.5 days vs 86 days). Previous clinical trial data have demonstrated that renal outcomes are better in patients initiating complement inhibitor treatment within 7 days of disease manifestation than in patients initiating treatment after 7 days [26]. Although, by the criteria of the study, 1 patient did not achieve a complete TMA response with ravulizumab, this patient responded to treatment and improved in all clinical parameters, including serum creatinine levels, normalization of platelets and LDH by Day 8, and complete recovery of renal function at last follow-up.

Regarding genetic analysis, we found no association between the identification of a pathogenic complement abnormality and response to ravulizumab (1 patient had a complement factor B (CFB) variant and 1 had anti-complement factor H (CFH) antibodies). The subgroup of patients analyzed here had complicated deliveries with significant bleeding, which could have triggered the syndrome, as suggested in other studies [8]. One patient with a severe predisposition (CFB pathogenic variant) had a normal delivery, whereas three patients with no identified pathogenic variants had severe bleeding complications, hypertension or pre-eclampsia, and consequently more severe clinical presentations. Five patients underwent emergency deliveries, 4 of which were by cesarean sections (a fifth patient also had a previously planned cesarean with complications postoperatively). Based on these observations in this subgroup and with only 2 patients testing positive for pathogenic variants or autoantibodies in our study, we hypothesize that patients with a severe genetic predisposition do not necessarily need a severe trigger to develop aHUS, whereas patients with a more severe trigger might not need an identified pathogenic variant in order to develop the disease. Nevertheless, all patients in this analysis had a severe presentation, regardless of genetic background and responded well to ravulizumab treatment.

The proportion of treatment-related AEs in the full 311 cohort was similar to that of the subgroup of patients in this analysis (34.5 and 37.5%, respectively). The current subgroup did not show a preponderance of any type of safety signal. In the full 311 cohort the most common AEs were headache, diarrhea, and vomiting [24], while headache and pyrexia were the most common in this subgroup. No patients died or contracted a meningococcal infection.

This study has limitations that must be noted. aHUS is an ultra-rare disease with an estimated prevalence of 4.9 per million and annual incidence rate between 0.23–1.9 per million [27]. As postpartum patients account for only around 4% of diagnosed cases of aHUS [3], there is not a large enough pool of complement inhibitor-naïve patients presenting postpartum to conduct a placebo-controlled trial. Currently this sub-analysis is the largest postpartum dataset utilized in a prospective study of patients with aHUS; no comparator or control group was utilized for the full 311 cohort, meaning that conclusions drawn from this subanalysis must be interpreted with caution. Further studies, if possible, with larger sample sizes, are required to fully confirm these findings.

## Conclusion

In this first prospective interventional trial assessing the efficacy and safety of the long-acting C5 inhibitor ravulizumab, TMA caused by aHUS was rapidly resolved in the subset of postpartum patients, with continued improvement over time and an acceptable safety profile. The results from this subset analysis suggest that ravulizumab is effective with a favorable safety profile in women presenting with aHUS postpartum.

## Supplementary Information

**Additional file 1: Supplementary Table S1.** Patients clinical profile.

**Additional file 2.** The PLS (Patient Lay Summary).

## Data Availability

Alexion will consider requests for disclosure of clinical study participant-level data provided that participant privacy is assured through methods like data de-identification, pseudonymization, or anonymization (as required by applicable law), and if such disclosure was included in the relevant study informed consent form or similar documentation. Qualified academic investigators may request participant-level clinical data and supporting documents (statistical analysis plan and protocol) pertaining to Alexion-sponsored studies. Further details regarding data availability and instructions for requesting information are available in the Alexion Clinical Trials Disclosure and Transparency Policy at http://alexion.com/research-development. Link to Data Request Form (https://alexion.com/contact-alexion/medical-information).

## Abbreviations

ADAMTS13: A disintegrin and metalloproteinase with a thrombospondin type 1 motif, member 13
AEs: Adverse events
aHUS: Atypical hemolytic uremic syndrome
BL: Baseline
CFB: Complement factor B
CFH: Complement factor H
CI: Confidence interval
eGFR: Estimated glomerular filtration rate
Esrd: End-stage renal disease
Hellp: Hemolysis, elevated liver enzymes and low platelets
HGB: Hemoglobin
ICU: Intensive care unit
IV: Intravenous
LDH: Lactate dehydrogenase
PE: Plasma exchange
PI: Plasma infusion
SAE: Serious adverse event
TEAE: Treatment-emergent adverse event
TESAE: Treatment-emergent serious adverse event
TMA: Thrombotic microangiopathy
TTP: Thrombocytopenic purpura
