# Correction to: Efficacy and safety of the long-acting C5 inhibitor ravulizumab in patients with atypical hemolytic uremic syndrome triggered by pregnancy: a subgroup analysis

**DOI:** 10.1186/s12882-021-02242-z

**Published:** 2021-02-02

**Authors:** Anja Gäckler, Ulf Schönermarck, Vladimir Dobronravov, Gaetano La Manna, Andrew Denker, Peng Liu, Maria Vinogradova, Sung-Soo Yoon, Manuel Praga

**Affiliations:** 1 Department of Nephrology, University Hospital Essen, University Duisburg-Essen, Essen, Germany; 2 Medizinische Klinik IV, LMU Klinikum, LMU, Munich, Germany; 3 Pavlov University, Research Institute of Nephrology, St. Petersburg, Russia; 4 Department of Experimental Diagnostic and Specialty Medicine (DIMES), Nephrology, Dialysis and Renal Transplant Unit, St. Orsola Hospital, University of Bologna, Bologna, Italy; 5 Alexion Pharmaceuticals, Inc., Boston, USA; 6 National Medical Research Centre for Obstetrics and Gynecology, Moscow, Russia; 7 Seoul National University Hospital, Seoul, Republic of Korea; 8 Instituto de Investigación Hospital Universitario 12 de Octubre i+12, Madrid, Spain

**Correction to: BMC Nephrol (2021) 22:5**

**https://doi.org/10.1186/s12882-020-02190-0**

Following publication of the original article [1], authors informed us that the PLS file was to be included in the supplementary information.

The original article [1] has been corrected.
