# Supplementary material for: Efficacy and safety of the long-acting C5 inhibitor ravulizumab in patients with atypical hemolytic uremic syndrome triggered by pregnancy: a subgroup analysis
Source: BMC Nephrol. 2021 Jan 6;22:5. doi: 10.1186/s12882-020-02190-0 (PMC7786907; doi:10.1186/s12882-020-02190-0)
Supplement: Supplementary file 1 — Additional file 1: Supplementary Table S1. Patients clinical profile. [file 12882_2020_2190_MOESM1_ESM.docx]

# Supplementary Materials

**Supplementary Table 1** Patients clinical profile

| **Patient** | **1** | **2** | **3** | **4** | **5** | **6** | **7** | **8** |
| --- | --- | --- | --- | --- | --- | --- | --- | --- |
| **Age, category** | 36–40 | 21–25 | 41–45 | 31–35 | 41–45 | 26–30 | 41–45 | 36–40 |
| **Prior pregnancies** | 1 | 0 | IVF multiple courses | 0 | 0 | 3 | 3 | 0 |
| **Genetic data** | CFB pathogenic variant (6:31950377_A/G, 1598A>G (p.Lys533Arg). Nucleotide changes are reported taking transcript NM_001710 as a reference). Central lab analysis by whole exome sequencing | CFH antibodies. No pathogenic variants identified. Central lab analysis by whole exome sequencing | No pathogenic variants identified. Analysis by individual center | No pathogenic variants identified. Analysis by individual center | Non-pathogenic heterozygous CFHR1/CFHR3 deletion. Analysis by individual center | Unknown | Non-pathogenic heterozygous CFHR1/CFHR3 deletion and heterozygous MCP-H2 haplotype. Analysis by individual center | No pathogenic variants identified. Analysis by individual center |
| **Clinical observations prior to TMA presentation and delivery** | Prior hypertension and on antihypertensives | New onset hypertension during pregnancy, stagnation of growth and suspicious fetal heartbeat CTG | IVF with twin pregnancy. Placental abruption | Renal failure and on antihypertensives | Pre-eclampsia, new-onset insulin dependent diabetes, arterial hypertension, elevated ALT, AST | Prior anemia, oliguria 2-3 days prior, antenatal fetal death | Gestational diabetes, placenta previa | Pre-eclampsia |
| **Time, delivery to diagnosis (days)** | 1 | 6 | 12 | 1 | 1 | 0 | 0 | 5 |
| **Delivery notes** | Vaginal delivery, TMA symptoms day 1 postpartum initially managed with PI and RBC transfusions | Urgent cesarean section due to suspicious fetal heartbeat, bleeding, hemorrhagic shock | Urgency cesarean section due to placental abruption; massive bleeding complications post hysterectomy, Initial TMA manifestations 3 days post delivery | Vaginal delivery with no complications; first TMA symptoms on day of delivery | Urgent cesarean section due to preeclampsia; intraabdominal hemorrhage, abruptio placentae, HELLP, hysterectomy 2 days after cesarean section | Urgent cesarean section due to fetal death, complete placental abruption | Planned cesarean section caused bleeding complications, resulting in patient needing multiple transfusions and hysterectomy | Urgent vaginal delivery due to pre-eclampsia |
| **Gestational age at delivery (weeks)** | 34 | >37 | 23 | 40 | 35 | 36-37 | 35 | 39 |
| **BP at TMA onset (mmHg) ^a^** | 153/96 | 165–200/80–100 | 120–160/70 | 160/100 | 160/100 | Normal BP noted | 163/88 | 179/90 |
| **Baby outcome** | Live birth | Live birth | Premature twins; 1 died immediately (brain halves had not separated) | Live birth | Live birth | Prenatal death | Live birth | Live birth |
| **Dialysis at baseline** | No | Started day -8 until day +3 | Started day -5 until day +9 | No | Started day -7 until day -5 | Started on day -1 until day -1 | Started day -5 until day +21 | No |
| **Dialysis at Day 183** | No | No | No | No | No | No | No | No |
| **Baseline eGFR (mL/min/1.73m^2^)^b^** | 18 | 10 | 10 | Missing | 10 | 10 | 10 | 12 |
| **eGFR at Day 183 (mL/min/1.73m)^b^** | 108 | 92 | 27 | 81 | 104 | 99 | 17 | 104 |
| **Baseline serum creatinine (μmol/L)^c^** | 273 | 277 | 693 | 469 | 51 | 465 | 758 | 352.5 |
| **Serum creatine at Day 183 (μmol/L)** | 54 | 69 | 181 | 72 | 55 | 62 | 268 | 57 |

^a^Blood pressure data were provided by individual treatment centers. ^b^eGFR was calculated using the Modification of Diet in Renal Disease formula; dialysis eGFR was set as an eGFR of 10 ml/min. ^c^When a patient was on dialysis at baseline, the first valid creatinine value used as the baseline value was the first assessment ≥6 days post-dialysis.

*BP* blood pressure, *CFB* complement factor B, *CFH* complement factor H, *CFHR1* complement factor H-related protein 1, *CFHR3* complement factor H-related protein 3, *CTG* cardiotocography, *eGFR* estimated glomerular filtration rate, *HELLP* hemolysis, elevated liver enzymes and low platelets, *IVF* in-vitro fertilization, *PI* plasma infusion, *RBC* red blood cell, *TMA* thrombotic microangiopathy

Baseline was defined as the period of screening up to before the point of the first study drug infusion, including Day 1.
