# Supplementary material for: Efficacy and safety of the long-acting C5 inhibitor ravulizumab in patients with atypical hemolytic uremic syndrome triggered by pregnancy: a subgroup analysis
Source: BMC Nephrol. 2021 Jan 6;22:5. doi: 10.1186/s12882-020-02190-0 (PMC7786907; doi:10.1186/s12882-020-02190-0)
Supplement: Supplementary file 2 — Additional file 2. The PLS (Patient Lay Summary). [file 12882_2020_2190_MOESM2_ESM.pdf]

Ravulizumab resolves blood counts and improves kidney function in women with atypical hemolytic uremic syndrome (aHUS) triggered by pregnancy

This is a summary of a clinical trial studying ravulizumab (ULTOMIRIS®), a new treatment for aHUS. This study included women who developed thrombotic microangiopathy (TMA) from aHUS following childbirth.

- Researchers have been studying ULTOMIRIS®, which is one of two approved treatments for aHUS (the other being eculizumab [SOLIRIS®]). ULTOMIRIS® was approved to treat aHUS in the USA in October 2019, and in the EU in June 2020. Researchers found that ULTOMIRIS® can be effective in adult patients with aHUS. Like SOLIRIS®, ULTOMIRIS® works by preventing the damage caused by an overactive complement system, reducing the burden of aHUS. ULTOMIRIS® is given only once every 8 weeks in adults, unlike SOLIRIS® which is given every 2 weeks.

What is aHUS?

- aHUS is a very severe disease caused by an overactive complement system. The complement system is part of the body's immune system. In patients with aHUS, the complement system becomes overactive and attacks the body's own cells, resulting in what is known as thrombotic microangiopathy (TMA). In TMA from aHUS, the lining of the body's small blood vessels become inflamed (swollen) and cause red blood cells to break, such that they stick together with platelets (small particles in the blood that help with clotting) and form tiny blood clots (also called thrombi) in the small blood vessels in the body. This results in hemolytic anemia, which is the destruction of red blood cells. Hemolytic anemia can be measured by a reduced number of red blood cells, broken red blood cells, elevated lactate dehydrogenase (LDH) levels in the blood, and fewer platelets. The multiple small blood clots lead to damage in the organs, most commonly in the kidneys, but they can also form in other organs. In addition to problems with kidney function, symptoms of aHUS can also include abdominal pain, swelling, seizures, fatigue, bruises, loss of consciousness and death.
- Researchers believe that for a person to develop aHUS, both a genetic abnormality in the complement system and a triggering condition are involved. The genetic abnormality causes an overactive complement system, and in combination with a triggering condition leads to aHUS. Pregnancy is one of the known triggering conditions in women with a genetic abnormality. aHUS triggered by pregnancy can occur at any point during pregnancy, but nearly 80% of patients that develop TMA caused by aHUS triggered by pregnancy do so shortly after childbirth.

What did this study investigate?

- Earlier studies have shown that SOLIRIS® helps improve the clincal outcomes for patients with aHUS.
- This study of ULTOMIRIS®, which was part of a larger study of patients with aHUS, was in a subgroup of patients who developed rapid and severe aHUS after childbirth. None of the patients had received ULTOMIRIS® before this study.
- This study investigated how well ULTOMIRIS® worked and how safe it was in women who had aHUS for the first time after childbirth.

What did this study find?

- Researchers studied eight women diagnosed with aHUS after childbirth. All women had medical emergencies associated with the pregnancy or delivery and received treatment with ULTOMIRIS® after being diagnosed. By the end of the study:
    - 87.5% of patients had a complete TMA response primary endpoint – see glossary
    - Platelet count and LDH levels returned to normal in 100% of patients
    - 100% of patients achieved a positive increase in hemoglobin levels
    - 100% of patients improved kidney function (monitored by estimated glomerular filtration rate [eGFR] – a measure of how well kidneys can filter the blood)
    - 100% of patients who were on dialysis due to poor kidney function at the beginning of the study were able to come off dialysis.
- The safety of ULTOMIRIS® was measured by looking at adverse events (see glossary). No unexpected adverse events were observed in this study, whose safety profile was consistent with the larger study of patients with aHUS.
- The most common adverse events in this study included headache and fever and were experienced by 37.5% of patients.

What are the main conclusions from this study?

- The results from this study indicate that treatment with ULTOMIRIS® resulted in rapid improvement in women who develop aHUS after childbirth.

- This summary is based on the research article by Dr Gäckler and colleagues, *Efficacy and safety of the long-acting C5 inhibitor ravulizumab in patients with atypical hemolytic uremic syndrome triggered by pregnancy: a subgroup analysis*.

Acknowledgments

- Alexion Pharmaceuticals, Inc. Boston, MA, USA sponsored this study. Alexion and authors would like to thank all patients and their families, physicians, and patient organizations for their assistance, participation, and support in this study. A patient representative for aHUS reviewed this summary, and the original authors of the full article reviewed and approved the summary.

Glossary

Adverse event An unwanted effect which happens while a patient is taking the drug. These are reported whether or not they are thought to be caused by the drug. The doctor then determines if the adverse event was related to the drug.

Complete TMA response In this study, the primary endpoint was 'complete TMA (thrombotic microangiopathy) response' defined by the percentage of patients who experienced simultaneous improvement in platelet count, LDH levels and serum creatinine levels (measures of kidney damage) at two separate assessments at least 4 weeks apart.

Primary endpoint A measure defined by regulatory authorities to use as a fixed measure of whether the study drug was effective.

Ravulizumab (ULTOMIRIS®) A drug developed by Alexion Pharmaceuticals, Inc. approved in the USA in 2019 and in the EU in 2020 for the treatment of aHUS.
